# A Cross-Sectional Study of Oral Hygiene Knowledge and Practices Among Undergraduate Dental Students: Bridging the Gap Between Theory and Practice

**DOI:** 10.7759/cureus.106569

**Published:** 2026-04-07

**Authors:** Vani Gupta, Agrima Baliyan, Noopur Kaushik, Parul Tyagi

**Affiliations:** 1 Department of Dentistry, Subharti Dental College and Hospital, Swami Vivekanand Subharti University, Meerut, IND; 2 Department of Pediatric and Preventive Dentistry, Subharti Dental College and Hospital, Swami Vivekanand Subharti University, Meerut, IND

**Keywords:** dental students, health, knowledge, oral hygiene, practices

## Abstract

Even though oral hygiene students receive formal education on oral hygiene protocols, there exists a wide gap between the theoretical and clinical application among dental students. This survey was cross-sectional and entailed undergraduate dental students, with the aim of testing their knowledge of oral hygiene practices and identifying the disparity between academic knowledge and real-life practices. Knowledge of oral hygiene principles, prevention strategies, and personal oral health practices, along with demographic information and self-reported oral health behaviours, was assessed using a validated questionnaire designed on the basis of structured questions.

The study established that although the students demonstrated strong oral hygiene principles at a theoretical level, their self-practice was not compatible with this theoretical knowledge. It was also found that there was a significant difference between perceived academic knowledge and its practical implementation in daily life, with determinants such as time constraints, level of awareness, and environmental factors contributing to the persistence of this gap.

Compliance among senior students was marginally better than that of junior students with respect to recommended practices. This study highlights the importance of addressing the theory-practice gap in dental training through the incorporation of more practical training, behaviour change teaching, and motivational interventions. Student compliance with best oral health practices can be improved through the inclusion of experiential learning and peer-driven programs.

These findings indicate that dental education programs should integrate behavioural components with theoretical knowledge to develop competent professionals who consistently practice and promote good oral health.

## Introduction

Oral health is an essential component of general health and well-being. Dental professionals play a critical role in promoting preventive oral health practices and educating patients about proper oral hygiene. Therefore, dental students are expected to possess adequate knowledge, positive attitudes, and appropriate behaviours related to oral health. Previous studies have emphasised the importance of assessing oral health knowledge and practices among dental students, as they will serve as future oral health educators and role models for the community [1].

Research has shown that the transition from preclinical to clinical training can influence students’ oral health awareness and personal practices. However, academic stress and demanding educational environments may also affect students’ health behaviours and coping strategies during their professional training [2]. Furthermore, variations in oral health behaviours have been reported among different groups of oral healthcare students, suggesting that educational exposure and training may significantly influence preventive practices [3].

Several studies have specifically evaluated the oral health knowledge, attitudes, and behaviours of dental students and reported varying levels of awareness and preventive practices among them [4]. Understanding these patterns is important for identifying gaps between theoretical knowledge and actual behaviour, thereby helping to improve educational strategies and promote better oral health practices among future dental professionals.

Dental students are expected to demonstrate exemplary oral hygiene practices, as they will serve as future oral health professionals and educators. Also, their personal attitudes and behaviours toward oral health can influence the quality of care they provide and the preventive advice they offer to patients [5].

Several studies have reported differences in oral health knowledge and behaviours between preclinical and clinical dental students [6-8]. However, many studies have also identified a gap between knowledge and actual practice among dental students. Further evidence of regional research patterns will strengthen the rationale [9].

Therefore, this study aimed to evaluate oral hygiene knowledge, attitudes, and behaviours among undergraduate dental students and to compare these factors between preclinical and clinical students.

Significance of the study

The research can have significant clinical practice, population health, and dental education implications. The findings will serve to support the existing literature on the subject of oral health behaviours among healthcare students, and this would translate to evidence-based solutions in the context of dental curriculum improvement. By identifying the barriers, it is possible to help educational institutions overcome knowledge application barriers and promote behaviour change among future dental professionals.

In addition, an understanding of factors that predispose oral health practices in dental students can be employed in the establishment of health promotion programs tailored to this special population. Lastly, healthy-mouthed dentists can perform better as upright role models to their patients and the community.

Literature review

Global Burden of Oral Diseases

The illnesses are extremely threatening to global well-being. Dental caries and periodontal disease, which were defined as the largest worldwide oral health burden, have always figured as the most crucial conditions affecting school-going children and the vast majority of adults in industrialised countries [5,6]. Further, it has been made clear that oral disease cannot be regarded as among the least common global health problems and that it carries a colossal socioeconomic burden that has been neglected in global health policy.

Almost one billion cases of oral disease contribute to the global burden, placing them nearly on par with the top five non-communicable diseases, according to the Global Oral Health Status Report 2022 of WHO [7].

Dental Students as Role Models

It is anticipated that these dental students will be future leaders in oral health care, so that they can serve as instructors on oral hygiene and guide oral care practices for their patients. As Mekhemar indicated, dentists should serve as good role models for patients regarding their oral behaviours, and past research has revealed how education can be useful in promoting oral health among dental students.

The preclinical-clinical transition is highly enlightening for students, as they no longer focus solely on learning about oral hygiene and patient care, but also on how to treat real patients and manage their oral health.

Oral Health Knowledge, Attitudes, and Practices (KAP) Among Dental Students

Al-Wesabi et al. (2019) conducted research in a privately owned Egyptian university and discovered that the scores on oral health knowledge, attitude, and behaviour were statistically different between preclinical and clinical dental students (p < 0.001). The research found that dental students in the final year showed a greater positive change than first-year students in terms of knowledge, attitude, and behaviour [4].

Yao et al. (2019) assessed the knowledge and oral health status of dental and medical undergraduate students at Sichuan University in China. The results showed that, although dental students recorded higher performance compared to medical students, both groups needed improvement in oral health status, behaviour, and knowledge. Notably, dental students in the first and third years, i.e., 56.8% and 40.3%, respectively, reported experiencing bleeding gums during brushing [9].

Ahamed et al. (2015) assessed preclinical and clinical pre-dental students’ oral health knowledge, attitude, and behaviour at Malabar Dental College, India. It was found that the difference between preclinical and clinical dental students was highly significant (p < 0.001). The research concluded that the role of dentists in teaching oral health to the population is extremely important, and that measuring knowledge, attitude, and behaviour at the student level is highly significant [1].

Tadin et al. (2022) conducted research on oral health knowledge and oral healthcare practices among healthcare and non-healthcare students at the University of Split in Croatia, involving 1,088 students. The findings demonstrated that oral health knowledge scores (median 13) were significantly higher among healthcare students (median 13) compared to non-healthcare students (median 11). The researchers pointed out that, more frequently, students who were more knowledgeable also employed additional aids to support oral hygiene [6].

Riad et al. (2022) assessed oral health knowledge, attitudes, and behaviour of German dental students using the Hiroshima University Dental Behavioural Inventory (HU-DBI). The authors concluded that the oral health attitudes and values of German dental students were mostly good compared to those in European, Middle-Eastern, and Asian countries. Preclinical students, in turn, had a higher (but statistically insignificant) HU-DBI mean score compared to the clinical students [10].

Preclinical vs. Clinical Dental Students

Mekhemar et al. (2020) carried out a pilot study on preclinical and clinical dental students, comparing the oral healthcare attitudes of the study subjects at Ain Shams University in Egypt. A survey conducted using HU-DBI on 149 dental students showed that, among clinical students (11.50) and preclinical students (10.63), the number of responses favouring oral health was high, as confirmed by statistical analysis (p < 0.05). The survey, however, showed a low group difference in enhancing oral hygiene behaviour and attitude among the subject groups [11].

Kassem et al. (2025) compared and contrasted the oral health attitudes of preclinical (187 students) and clinical (144 students) dental students at Cordoba Private University, Syria. In some areas of change, such as practising regular brushing (p = 0.001), professional brushing (p = 0.001), and reduced intake of sweets (p = 0.003), clinical students showed superiority over preclinical students. Nonetheless, with respect to bad habits, the tendency to smoke appeared more frequently during the clinical years [12].

Mohan et al. (2024) compared the oral health knowledge, attitudes, and behaviour of preclinical and clinical dental students in a single Indian institution. The research results showed that clinical dental students exhibited marginally higher KAP on oral health than preclinical students, which could be a sign of challenges in the transition between preclinical and clinical stages. Females were more oriented toward oral health in comparison with males in intergender comparisons [8].

Arandi (2025) used the HU-DBI to establish the level of knowledge, attitude, and oral health behaviour regarding oral health among dental students at Arab American University, Palestine. There was a statistically significant difference in total scores (7.70 vs. 6.68; p = 0.0001), knowledge (3.60 vs. 3.25; p = 0.0168), and behaviour domains (2.46 vs. 1.65; p = 0.0001) between clinical and preclinical students. The overall HU-DBI scores (7.54 ± 1.71 vs. 6.83 ± 1.77) for female students during dental studies were significantly higher than those of male students (p = 0.0001) [13].

Cross-Cultural Comparisons

Komabayashi et al. (2005) applied HU-DBI to conduct a cross-national survey on dental students at the University of Leeds, Britain, and West China University of Medical Sciences, China. Its major findings included the following: self-reported gingival bleeding was more common among the Chinese students; 29% of the Chinese students believed they would wear dentures when they were old, as opposed to 7% of the British students; and 54% of the Chinese students only visited the dentist when they had symptoms, as opposed to 13% of the British students [14].

Al-Wahadni et al. (2004) compared dental students and dental technology/dental hygiene students in Jordan in terms of oral health behaviour. Students in the other disciplines exhibited some significant differences. The dental technology/dental hygiene students were found to be more knowledgeable about bleeding gums during brushing compared to the dental students [3].

Jaramillo et al. (2013) utilised HU-DBI to compare the oral health attitudes and behaviour of dental and civil engineering students in Colombia. The outcomes of dental education were reported to be higher in comparison to those of civil engineering students, and they were used in the process of modifying the dental school curriculum to enhance oral health attitudes and behaviour [7].

Barriers and Stress Factors

Tariq et al. (2024), to determine the relationship between health behaviours, perceived stress, self-efficacy, and oral hygiene practices, used a national survey of 904 dental students in Pakistan. The authors discovered that dentistry students are exposed to the rigorous burden of the course program, which may result in incompetence and stress, negatively affecting self-efficacy and contributing to the neglect of healthy practices, including oral health [15].

Alzahem et al. (2011) subdivided other stress-causal factors among dental students into five categories: living accommodation, educational environment, personal, academic, and clinical factors. Scheduling of exams, manual dexterity and evaluation, neighbourhood and research needs, and clinical and preclinical placements can also cause stress among students in the dentistry profession [5].

Al-Sowygh (2013) opined that the extent of academic distress and perceived stress presented a matter of concern for dental students in Saudi Arabia and thus required specific preventive intervention. It is established that two-thirds of dental undergraduate students were reported to be under stress [2].

Long et al. (2024) carried out a scoping review of resiliency in oral health professional training and found that elements in academics and clinical work were the most prevalent causes of stress in dental students. A shift in a clinical setting is especially stressful, and prolonged stress can lead to physical and mental burnout [16].

Research Gap

However, irrespective of the increasing literature on oral health KAPs among dental students, there are some gaps. Although the differences reported between knowledge and practice in different categories of people have been addressed in studies, no research has specifically targeted the factors that hinder dental students from translating their theoretical knowledge into personal oral hygiene practices. In addition, the majority of available research has been based on a single organisation or a particular area of study and thus cannot be generalised. Research is also needed to investigate the importance of curriculum design and learning interventions in closing the theory-practice gap.

Secondly, despite extensive use of the HU-DBI in the analysis of oral health behaviour, there is a lack of literature combining this standardised measure with qualitative analysis of the factors that contribute to low rates of oral hygiene behaviour among dental students. Such gaps will be addressed in the current study, which will quantify the level of knowledge and practices of oral hygiene among undergraduate dental students, take a holistic view, determine some of the barriers to applying knowledge, and provide evidence-based suggestions on how the curriculum can be enhanced.

Research Objectives

The objectives are as follows: (i) To determine self-reported oral hygiene behaviour, such as how frequently one brushes their teeth, the type of brushing, the use of interdental cleaning aids, and how often one visits the dentist; (ii) to determine the knowledge and oral hygiene practices of preclinical and clinical dental students; (iii) to determine the disconnection between assumed and actual oral health practices among dental students.

Research Questions

The research questions are as follows: (i) What is the oral hygiene knowledge of undergraduate dental students? (ii) What is the performance of undergraduate dental students in their oral health behaviour? (iii) Do preclinical and clinical dental students differ in their knowledge and oral hygiene practices?

## Materials and methods

Research design

This study employed a quantitative, descriptive, cross-sectional survey design. Its purpose was to assess the KAP of oral hygiene among undergraduate dental students and to identify the theory-practice gap using self-reported information in real time.

Study setting

The research was carried out among a group of undergraduate dental students at Subharti Dental College and Hospital, Swami Vivekanand Subharti University, Meerut, India. Information was gathered using an online Google Form, titled Bridging the Gap between Theory and Practice, which included question-based items to assess oral hygiene knowledge and behaviour. Ethical approval was obtained from the University Ethics Committee (approval no. SMC/UECM/2025/1399).

Study population

The sample was composed of dental undergraduate students taking the BDS course, covering both preclinical and clinical stages.

Inclusion Criteria

The inclusion criteria of this study were: (i) all students enrolled in BDS (first year to internship); (ii) respondents who completed the online questionnaire voluntarily.

Exclusion Criteria

The exclusion criteria of this study were: (i) students from non-dental programs; and (ii) incomplete or blank responses (none were found in the final dataset).

Sample size

The final dataset contained a total of 422 responses. All responses were complete and valid. Thus, the final sample size was 422 participants (n = 422). This large sample enhances statistical validity and reliability.

Sampling technique

Convenience sampling was used due to the distribution of the questionnaire link in academic groups and through social media. Students who volunteered to participate did so of their own free will. This approach is suitable for online, cross-sectional research in the academic sector.

Research instrument

Actual data were used to create a structured, self-administered Google Form questionnaire (see Appendix 1), containing 21 variables from which data were collected. The questionnaire included both closed-ended and multiple-choice questions, ensuring ease of analysis.

Validity and reliability

The questionnaire items were constructed using a knowledge base validated by previous KAP studies. Faculty members reviewed the content to ensure content validity. Pilot testing was conducted on a small group of participants, and internal reliability was assessed. The tool exhibited satisfactory internal consistency, with a Cronbach’s alpha above the acceptable reliability level of 0.70.

Data collection procedure

The institution provided ethical approval and permission before commencing the study. The link to the Google Form was shared via official student groups, WhatsApp groups, and classroom networks to facilitate participation. Involvement was voluntary and anonymous. Responses were automatically recorded in an Excel file (Microsoft® Corp., Redmond, WA, USA), and the dataset of 422 completed responses was then exported for statistical analysis.

Data analysis

The information in the Excel file was transferred to Microsoft Excel and IBM SPSS Statistics for Windows, Version 28 (Released 2021; IBM Corp., Armonk, NY, USA). The statistical methods were as follows: (i) descriptive statistics - frequencies, percentages, mean, and standard deviation (where applicable); (ii) comparative analysis - preclinical vs. clinical differences in knowledge and practice, chi-square tests for categorical variables, and t-tests for mean differences (if applicable); (iii) knowledge-practice gap assessment - comparison between ideal knowledge (e.g., twice-a-day brushing) and actual behaviours recorded in the dataset.

Ethical considerations and limitations

This study carefully considered ethical issues. All subjects were informed about the study, and participation was completely voluntary, as stated at the beginning of the Google Form. Respondents were not asked to provide any personal information, ensuring that no personal data was collected. Data confidentiality was strictly maintained during the study, and the data were used solely for academic and research purposes.

This study was conducted with careful consideration of ethics. All participants were informed about the study and provided informed consent at the start of the Google Form, and participation was fully voluntary. No personal details of the respondents were collected, ensuring their anonymity. Data confidentiality was also maintained throughout the research, and the data collected were used solely for academic and research purposes.

## Results

Demographic characteristics of participants

This was a cross-sectional study involving 422 undergraduate dental students. Table 1 shows the demographic variables of the respondents. The majority of participants were aged 18-24 years (80.3%) and 25-30 years (13.5%), with those above 35 years comprising 2.8%. In terms of year of study, first-year students were the largest group (37.0%), followed by interns (16.1%), and others, such as working professionals (12.3%) and second-, third-, and fourth-year students (10.9%-12.1%). To compare the results, participants were divided into preclinical (first and second year; n = 207, 49.1%) and clinical stages (third year, fourth year, and interns; n = 163, 38.6%). The remaining 52 respondents (12.3%) belonged to working professionals and other categories.

**Table 1:** Demographic Characteristics of Participants (N = 422)

Variable	Frequency (n)	Percentage (%)
Age Group		
18-24 years	339	80.3
25-30 years	57	13.5
31-35 years	14	3.3
>35 years	12	2.8
Year of Study		
1st year	156	37.0
2nd year	51	12.1
3rd year	46	10.9
4th year	49	11.6
Interns	68	16.1
Others	52	12.3
Academic Status		
Preclinical (1st & 2nd year)	207	49.1
Clinical (3rd, 4th year & interns)	163	38.6
Others	52	12.3

Assessment of oral hygiene knowledge

Table 2 shows the distribution of responses to the questions on oral hygiene knowledge among dental students. A majority of the students (83.6%) correctly answered that teeth should be brushed twice a day. Regarding brushing time, 57.8% of respondents reported one to two minutes, and 36.5% reported two to three minutes as the recommended duration for brushing. Most respondents (71.6%) knew that a soft-bristled toothbrush is recommended, while 27.0% indicated a medium-bristled type.

**Table 2:** Distribution of Oral Hygiene Knowledge Responses (N = 422) *Correct/Recommended response based on evidence-based guidelines

Knowledge Item	n	%
Recommended Brushing Frequency		
Twice a day*	353	83.6
Once a day	59	14.0
After every meal	10	2.4
Ideal Brushing Duration		
1-2 minutes	244	57.8
2-3 minutes*	154	36.5
Less than 1 minute	19	4.5
More than 5 minutes	5	1.2
Recommended Bristle Type		
Soft*	302	71.6
Medium	114	27.0
Hard	6	1.4
Brushing Technique Knowledge		
Modified bass technique*	230	54.5
Horizontal scrub method	139	32.9
Fones technique	35	8.3
Charter's technique	15	3.6
Bass technique	3	0.7
Toothbrush Replacement Frequency		
Every 3 months*	279	66.1
Every 1 month	74	17.5
Every 6 months	65	15.4
Once a year	4	0.9
Recommended Flossing Frequency		
Once a day*	169	40.0
Twice a week	115	27.3
Once a week	104	24.6
Only when food is stuck	33	7.8

For brushing technique, 54.5% of respondents cited the modified bass technique as the most recommended method, followed by the horizontal scrub method (32.9%). Regarding toothbrush replacement, 66.1% correctly answered every three months, and 17.5% answered every month. For flossing frequency, 40.0% correctly identified once daily as the recommended frequency.

Composite knowledge score analysis

**Figure 1:**
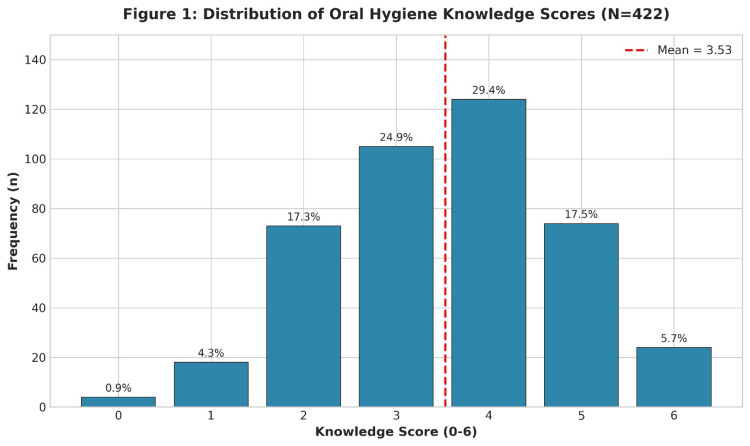
Distribution of Oral Hygiene Knowledge Scores

The composite knowledge score was obtained by referring to six evidence-based parameters of oral hygiene (range: 0-6). The mean knowledge score was 3.53 (rounded to 4.0), with a standard deviation of 1.29. Table 3 shows the histogram of knowledge scores among the study population. The distribution of respondents’ knowledge scores is shown in Figure 1, illustrating a concentration of scores between 3 and 4, with a mean of 3.53.

**Table 3:** Distribution of Knowledge Scores (N = 422)

Score	Frequency (n)	Percentage (%)
0	4	0.9
1	18	4.3
2	73	17.3
3	105	24.9
4	124	29.4
5	74	17.5
6	24	5.7
Mean ± SD	3.53 ± 1.29	

Comparison of oral hygiene knowledge: preclinical vs. clinical students

Table 4 presents a comparison of correct responses on knowledge between preclinical (n = 207) and clinical (n = 163) dental students. Chi-square tests were conducted to establish the statistical significance of the differences between the two groups.

**Table 4:** Comparison of Correct Knowledge Responses Between Preclinical and Clinical Students **p < 0.01; ***p < 0.001 (Chi-square test)

Knowledge Item	Preclinical %	Clinical %	p-value
Brushing twice daily	84.1	89.0	0.370
Brushing 2-3 minutes	44.9	28.8	0.010**
Soft bristles recommended	71.0	77.3	0.236
Modified bass/bass technique	43.0	79.8	<0.001***
Replace the brush every 3 months	61.8	73.6	0.005**
Floss once daily	39.1	46.0	0.418

Significant differences were found in knowledge about brushing technique (χ² = 55.294, p = 0.001), frequency of brushing and toothbrush replacement (χ² = 12.882, p = 0.005), and ideal duration of brushing (χ² = 11.395, p = 0.010). Interestingly, students in the clinical group were much more knowledgeable about the modified bass technique (79.8% vs. 43.0%) than their preclinical counterparts. However, preclinical students were more knowledgeable about optimal brushing time (44.9% vs. 28.8%).

Table 5 further illustrates this difference, showing that preclinical students were more knowledgeable about optimal brushing time (44.9% vs. 28.8%).

**Table 5:** Comparison of Knowledge Scores Between Preclinical and Clinical Students

Group	n	Mean	SD	Median
Preclinical	207	3.44	1.26	3.0
Clinical	163	3.94	1.17	4.0

**Figure 2:**
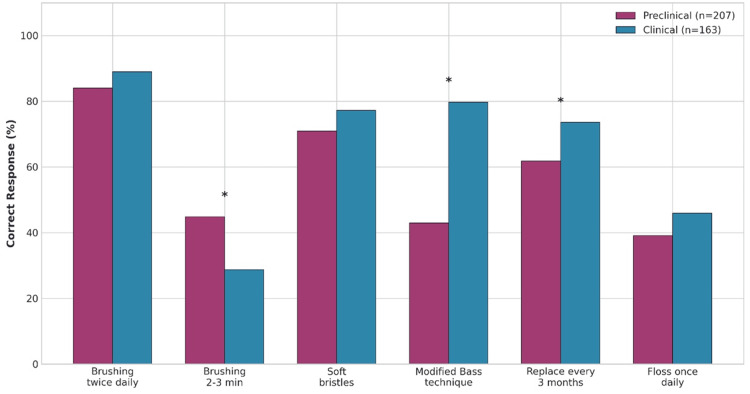
Comparison of Correct Knowledge Response Between Preclinical and Clinical Student

**Figure 3:**
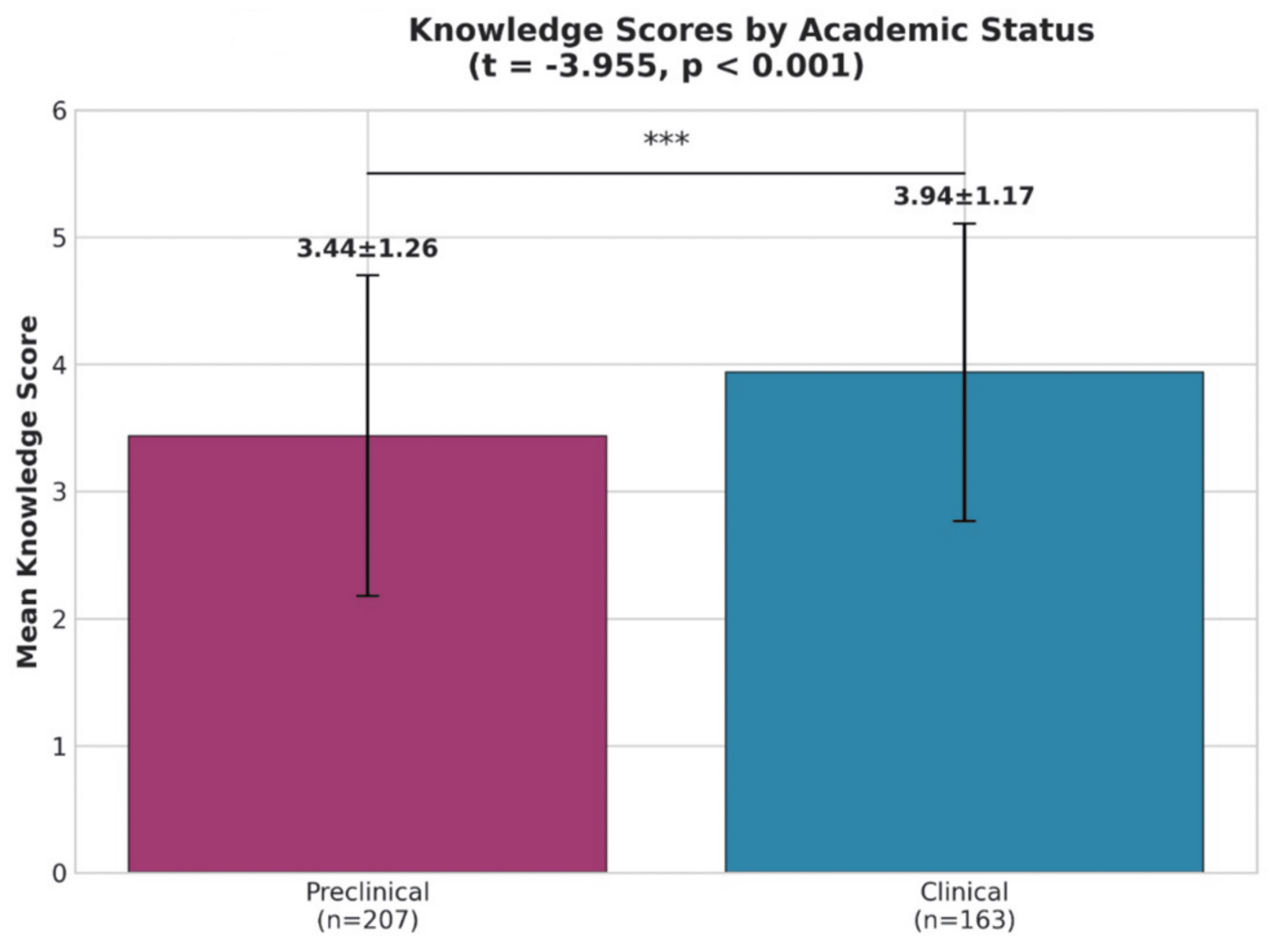
Knowledge Scores by Academic Status

The comparison of mean knowledge scores between preclinical and clinical participants is illustrated in Figure 2, showing significantly higher scores among clinical participants (3.94 ± 1.17) compared to preclinical participants (3.44 ± 1.26) (p < 0.001). Clinical students exhibited a mean knowledge score that was significantly greater (M = 3.94, SD = 1.17) than that of preclinical students (M = 3.44, SD = 1.26). This difference was statistically significant, with both parametric (t = -3.955, p = 0.001) and non-parametric (U = 12,590.0, p = 0.001) tests confirming significance (Figure 3).

Knowledge scores by year of study

ANOVA was performed using one-way analysis to test the differences in knowledge scores between different years of study. The results showed that the differences between groups were statistically significant (F = 5.351, p < 0.001).

**Figure 4:**
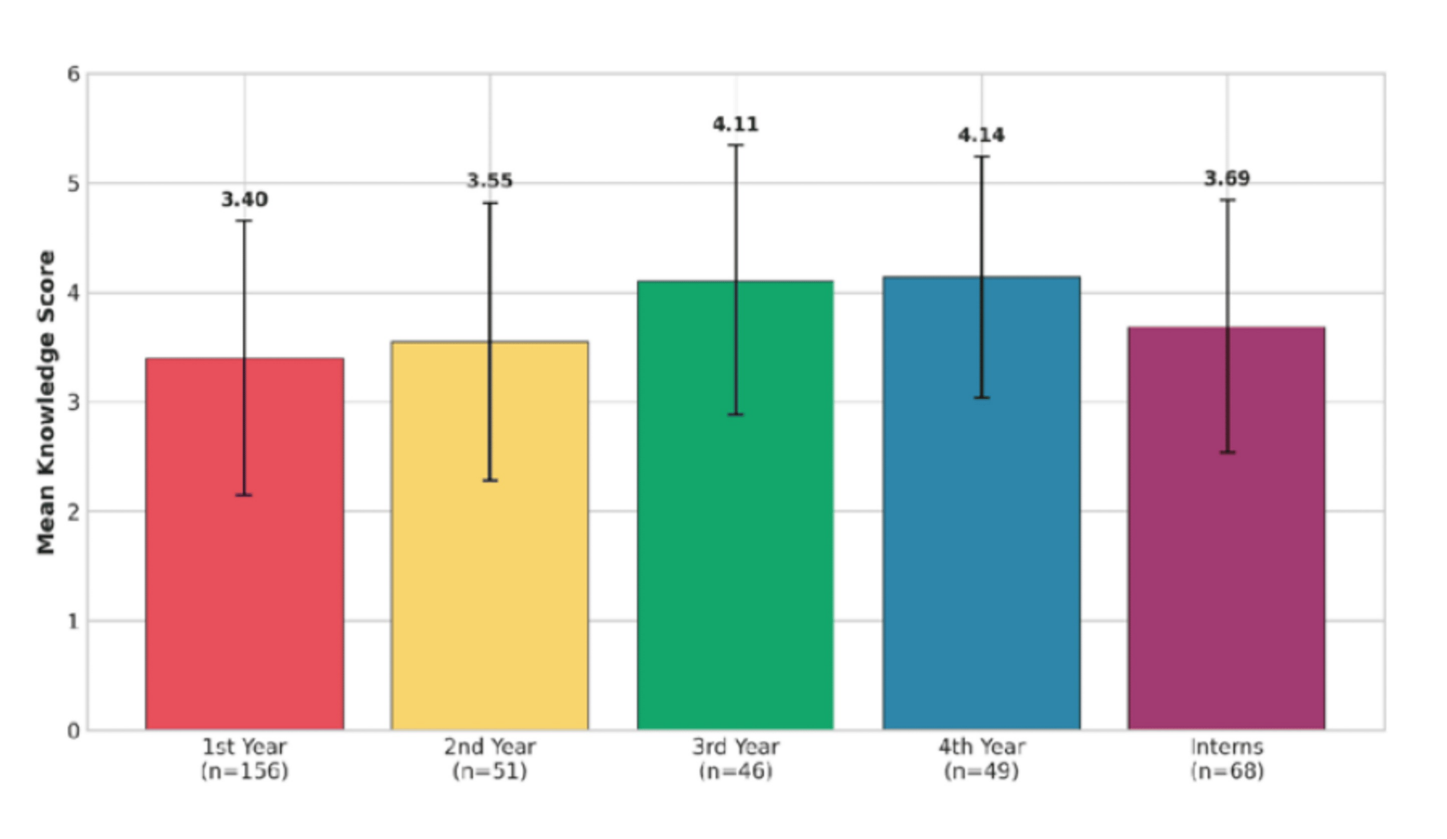
Knowledge Scores by Year of Study (One-Way ANOVA: F = 5.351, p < 0.001)

As shown in Table 6, the differences between groups were statistically significant (F = 5.351, p < 0.001). The distribution of mean knowledge scores across different academic years and internship status is shown in Figure 4, with higher mean scores observed among third-year (4.11) and fourth-year students (4.14), followed by interns (3.69), and lower scores among first-year (3.40) and second-year students (3.55).

**Table 6:** Knowledge Scores by Year of Study

Year of Study	N	Mean	SD
1st year	156	3.40	1.25
2nd year	51	3.55	1.27
3rd year	46	4.11	1.23
4th year	49	4.14	1.10
Interns	68	3.69	1.15

Tukey HSD tests showed statistically significant differences between first-year and third-year students (p = 0.006) and between first-year and fourth-year students (p = 0.002). Fourth- and third-year students had the highest average knowledge scores (M = 4.14 and 4.11, respectively), while the lowest score of 3.40 was obtained by first-year students. Interestingly, the interns had a slightly lower score (M = 3.69) than the third- and fourth-year students, but the difference was not significant.

Attitudes toward oral hygiene

Table 7 shows the distribution of attitudes towards oral hygiene among the study participants. The vast majority (97.2%) of students believed, or strongly believed, that oral hygiene is directly linked to overall health. Similarly, 96.9% considered dental awareness camps essential or very essential for maintaining their oral health.

**Table 7:** Distribution of Attitudes Toward Oral Hygiene (N = 422)

Attitude Item	n	%
Oral Hygiene Affects General Health		
Strongly agree	267	63.3
Agree	143	33.9
Neutral	11	2.6
Disagree	1	0.2
Importance of Dental Awareness Camps		
Very important	324	76.8
Important	85	20.1
Somewhat important	11	2.6
Not important	1	0.2
Regular Mouthwash Use for Good Oral Health		
Strongly agree	137	32.5
Agree	161	38.2
Neutral	89	21.1
Disagree	34	8.1

The chi-square test showed a statistically significant difference in the attitude toward regular mouthwash use between preclinical and clinical students (χ² = 13.928, p = 0.008). Clinical students expressed more disagreement (13.5% vs. 3.9%) regarding regular mouthwash use, indicating greater evidence-based knowledge of mouthwash recommendations.

Self-reported oral hygiene practices

Table 8 depicts the self-reported oral hygiene behaviour of dental students. With regard to dental check-ups, 50.9% of the students reported visiting a dentist in the last six months, and 20.1% indicated that they had visited a dentist 6-12 months ago. Regarding mouthrinse use, 26.1% used mouthrinse daily, while 24.6% did not use mouthrinse at all.

**Table 8:** Distribution of Self-Reported Oral Hygiene Practices (N = 422)

Practice Item	N	%
Last Dental Check-up		
Within past 6 months	215	50.9
6-12 months	85	20.1
1 year ago,	59	14.0
More than 2 years ago	53	12.6
Never	10	2.4
Type of Toothbrush Used		
Manual	388	91.9
Electric-oscillating	18	4.3
Electric-vibrating	13	3.1
Other	3	0.7
Mouthrinse Usage Frequency		
Daily	110	26.1
Once a week	103	24.4
Only when needed	104	24.6
Don't use mouthrinse	104	24.6
Professional Cleaning Recommendation		
Once in 6 months	266	63.0
6-12 months	88	20.9
Once a year	61	14.5
Never	5	1.2

Comparison of oral hygiene practices: preclinical vs. clinical students

**Figure 5:**
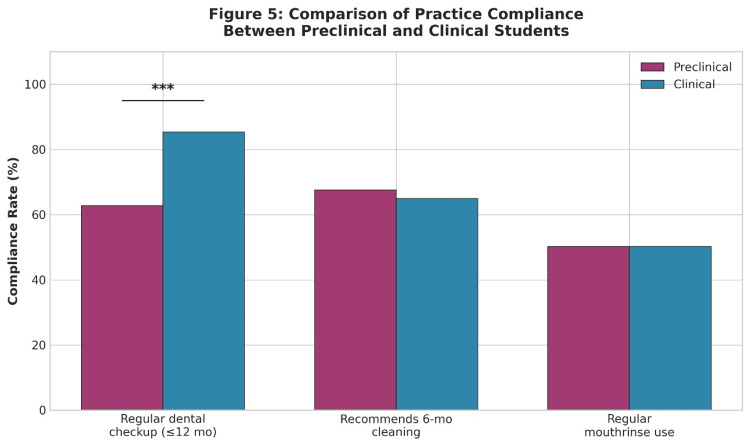
Comparison of Practice Compliance Between Preclinical and Clinical Students

Table 9 demonstrates the comparison of compliance with oral hygiene practices between preclinical and clinical students. The proportion of clinical students (85.3%) who reported visiting a dentist within the past 12 months was significantly higher than that of preclinical students (62.8%) (χ² = 22.090, p = 0.001). As shown in Figure 5, clinical participants exhibited significantly higher compliance with regular dental check-ups compared to preclinical participants.

**Table 9:** Comparison of Practice Compliance Between Preclinical and Clinical Students ***p < 0.001 (Chi-square test)

Practice Item	Preclinical %	Clinical %	p-value
Regular dental checkup (≤12 months)	62.8	85.3	<0.001***
Recommends 6-month professional cleaning	67.6	65.0	0.678
Regular mouthrinse use	50.2	50.3	1.000

Independent samples t-tests showed that students with higher practice compliance demonstrated a significant difference in mean practice compliance (M = 2.01, SD = 0.90) (t = -2.064, p = 0.040).

Assessment of the theory-practice gap

**Figure 6:**
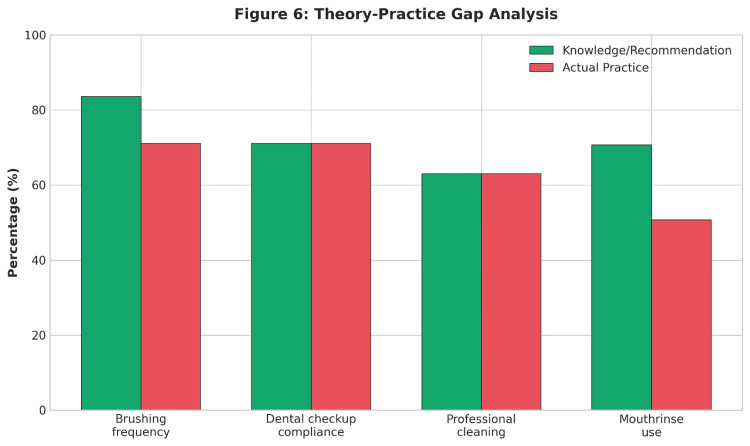
Theory-Practice Gap Analysis

Table 10 provides a gap analysis between theoretical knowledge and actual practice among dental students. Although knowledge scores in some areas were high, compliance with practice was remarkably lower, suggesting a wide theory-practice gap. As shown in Figure 6, actual oral health practices lagged behind reported knowledge and recommended behaviours.

**Table 10:** Theory-Practice Gap Analysis

Domain	Knowledge %	Practice %	Gap
Brushing twice daily	83.6	-	-
Regular dental checkup compliance	-	71.1	-12.5
Professional cleaning every 6 months	63.0	63.0	0.0
Daily flossing	40.0	-	-

The correlation analysis revealed a weak and non-significant relationship between overall knowledge scores and practice scores (Spearman r = 0.057, p = 0.241; Pearson r = 0.078, p = 0.111), i.e., the higher the theoretical knowledge, the higher the practical behaviours. However, attitude and practice scores showed a moderate positive relationship (r = 0.275, p < 0.001).

**Figure 7:**
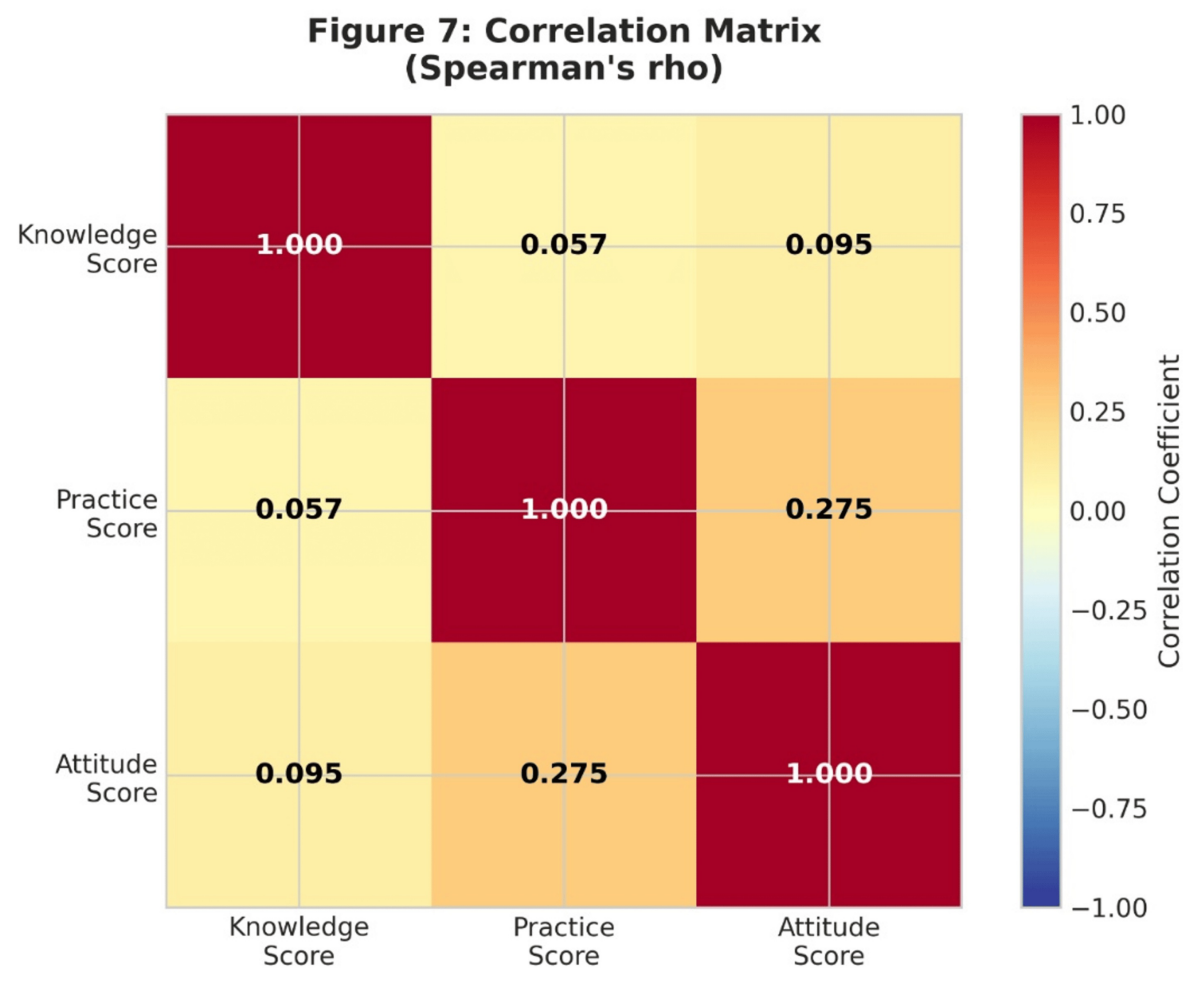
Correlation Matrix (Spearman's Rho)

Attitude and practice scores exhibited a moderate positive correlation (r = 0.275, p < 0.001), while correlations involving knowledge were weak (Table 11). These associations are illustrated in Figure 7.

**Table 11:** Correlation Matrix of Knowledge, Attitudes, and Practice Scores ***p < 0.001 (Spearman's rho)

Variable	Knowledge	Practice	Attitude
Knowledge score	1.000	0.057	0.095
Practice score	0.057	1.000	0.275***
Attitude score	0.095	0.275***	1.000

Willingness for future participation in awareness programs

When questioned about their readiness to participate in future oral hygiene awareness campaigns, most students (77.0%) gave a positive response, suggesting a high level of openness to further educational campaigns. Additionally, 17.8% said they were undecided (“Maybe”), and only 5.2% indicated that they were not interested in getting involved [17,18].

Summary of key findings

The summary of key findings is as follows: (i) The average knowledge score was 3.53 ± 1.29 (range: 0-6), with 83.6% of students correctly identifying the frequency of brushing, but only 36.5% identifying the correct duration of brushing. (ii) Clinical students showed a considerably higher knowledge score (3.94 ± 1.17) compared to preclinical students (3.44 ± 1.26), p < 0.001. (iii) Knowledge differences were also significant between preclinical and clinical years regarding brushing technique; the modified bass technique was known by 43.0% of preclinical and 79.8% of clinical students, respectively. (iv) Knowledge and practice scores were not significantly related (r = 0.057), indicating a large gap between theory and practice. (v) Compliance with dental check-ups was significantly higher in clinical students (85.3% vs. 62.8%, p < 0.001) compared to preclinical students. (vi) The correlation between attitude scores and practice scores was moderate (r = 0.275, p < 0.001), suggesting that attitude scores may be a better predictor of behaviour than knowledge [19].

## Discussion

The present study evaluated oral hygiene knowledge, attitudes, and behaviours among undergraduate dental students and compared differences between preclinical and clinical students. The findings revealed that, although dental students generally possessed adequate theoretical knowledge regarding oral health, this knowledge was not always reflected in their personal oral hygiene practices. This indicates a persistent gap between knowledge and behaviour among dental students.

In the current study, clinical students demonstrated higher knowledge scores compared with preclinical students. This finding is consistent with previous research conducted by Ahamed et al. (2015), which reported that clinical dental students exhibited better oral health knowledge and behaviours due to increased clinical exposure and training [1]. Similarly, Mekhemar et al. (2020) reported improved oral health attitudes among clinical students, suggesting that clinical training and patient interaction contribute positively to students’ awareness and preventive practices [11].

Despite the relatively high knowledge levels observed in the present study, several behavioural inconsistencies were identified. While a majority of students were aware that brushing should be performed twice daily, a considerably smaller proportion knew the recommended brushing duration. This discrepancy indicates that, although students possess general knowledge of oral hygiene practices, they may lack a detailed practical understanding or fail to consistently apply recommended guidelines.

These findings align with the results of a study conducted by Yao et al. (2019), which demonstrated that dental students often exhibit better theoretical knowledge compared with students from other healthcare disciplines but may still demonstrate suboptimal oral hygiene practices [9]. Similarly, Riad et al. (2022) reported that, although dental students showed positive attitudes toward oral health, behavioural adherence to preventive measures remained inconsistent [10].

The present study also observed that clinical students reported higher compliance with routine dental check-ups compared with preclinical students. This observation is in agreement with findings reported by Kassem et al. (2025), who found that clinical students were more likely to engage in preventive dental visits and maintain improved oral hygiene practices [12]. Increased exposure to patient care and clinical training may enhance students’ understanding of the importance of preventive oral healthcare.

Another important finding of the study was the weak correlation between oral health knowledge and oral hygiene practices. This suggests that possessing knowledge alone may not be sufficient to influence behavioural change. Similar observations have been reported by Komabayashi et al. (2006), who highlighted that psychological factors, personal motivation, and cultural influences may also affect oral health behaviours among dental students [20].

Stress and academic workload may also play a role in influencing students’ oral health behaviours. Previous studies have identified dental education as a demanding academic environment that can contribute to stress and unhealthy lifestyle habits among students. Alzahem et al. (2011) reported that high levels of academic stress among dental students may negatively influence their personal health behaviours, including oral hygiene practices [5]. Furthermore, resilience and coping mechanisms among dental students have been highlighted as important factors affecting health behaviours during professional training [16]. Also, exposure to dental misinformation and social media influences has been shown to shape health behaviours and may partly explain discrepancies between knowledge and practice among students [21].

The findings of this study highlight the need for educational strategies that extend beyond theoretical instruction. Incorporating behavioural training, preventive dentistry modules, and self-care awareness programs into dental curricula may help encourage students to adopt healthier oral hygiene habits. Promoting self-awareness among dental students is particularly important, as they are expected to serve as role models for their patients and communities. Therefore, dental education plays an important role in shaping oral health behaviours among students, improving oral health awareness and preventive practices, while global policies emphasise strengthening preventive oral healthcare systems [22-24].

## Conclusions

The present study highlights a noticeable gap between theoretical knowledge and actual oral hygiene practices among undergraduate dental students. Although the majority of participants demonstrated adequate knowledge regarding oral health and preventive practices, this knowledge was not always consistently reflected in their personal oral hygiene behaviours. Clinical students exhibited relatively higher knowledge levels and better oral hygiene practices compared with preclinical students, suggesting that clinical exposure and patient interactions may positively influence students’ attitudes and behaviours toward oral health. However, the weak correlation observed between knowledge and practice indicates that theoretical understanding alone may not be sufficient to promote behavioural change.

These findings emphasise the need for dental education programs to incorporate strategies that promote behavioural modification alongside theoretical learning. Integrating preventive dentistry training, practical demonstrations, and self-care awareness programs within the dental curriculum may help bridge the gap between knowledge and practice. Encouraging dental students to adopt and maintain optimal oral hygiene behaviours is essential, as they will serve as future oral health professionals and role models responsible for promoting oral health awareness within the community.

## Appendices

Appendix 1

Section A: Demographic Variables

Year of study

Age group

Academic status

Section B: Oral Hygiene Knowledge

Based on real dataset variables

Recommended brushing frequency

Ideal brushing duration

Use of fluoridated toothpaste

Awareness about interdental cleaning

Knowledge about dentist visit intervals

Section C: Oral Hygiene Practices

Real dataset includes

Actual brushing frequency

Time spent brushing

Type of toothbrush and toothpaste

Use of mouthwash/interdental aids

Symptoms such as bleeding gums

Frequency of replacing toothbrush

Last dental visit

Section D: Attitudes & Awareness

Perception of own oral hygiene

Willingness to participate in awareness programs
